# Simulating the distribution changes and future climate responses of a key invasive plant in Northwest China’s natural grasslands using an optimized MaxEnt model

**DOI:** 10.3389/fpls.2026.1858197

**Published:** 2026-07-09

**Authors:** Gulsum Rixit, Rukeya Sawut, Alimujiang Kasimu, Ainiwaer Aimaier, Ma Bao Yue

**Affiliations:** 1 College of Geographic Science and Tourism, Xinjiang Normal University, Ürümqi, China; 2 Laboratory of Arid Zone Lake Environment and Resources, Xinjiang Normal University, Ürümqi, China; 3 Navigation College, Dalian Maritime University, Dalian, China; 4 Xinjiang Uygur Autonomous Region Academy of Animal Sciences, Grassland Research Institute, Ürümqi, China; 5 Xinyuan County Forestry and Grassland Bureau, Yili, China

**Keywords:** climate change, kuenm optimization, MAXENT model, ragweed, suitable habitat distribution, Xinyuan County

## Abstract

**Background:**

Global biodiversity and ecosystem services are threatened by invasive alien plants. Ragweed (Ambrosia artemisiifolia L.), a globally problematic weed, has rapidly spread across Xinjiang's Ili Prefecture—particularly in Xinyuan County—and presents serious obstacles to local agriculture, animal husbandry, and human health. However, in the context of climate change, the fine-scale distribution dynamics and key driving mechanisms of this species remain poorly understood.

**Methods:**

Based on field survey data from Xinyuan County, a Kuenm-optimized MaxEnt model was combined with four shared socioeconomic pathway (SSP) scenarios from CMIP6 to systematically simulate the spatiotemporal evolution of the potential habitat of ragweed at present and from the 2030s to the 2090s.

**Results:**

(1) The optimized model exhibited exceptional predictive accuracy (AUC = 0.991; Partial ROC ratio = 1.959, p < 0.001; empirical omission rate = 0.0267), with precipitation seasonality (BIO15) and isothermality (BIO3) as the primary environmental factors constraining ragweed distribution. (2) Current high-risk zones predominantly exhibit distributions along river valley alluvial plains and road networks. Overlay analysis revealed that mountain steppe grasslands face the most severe stress, followed by low–middle mountain meadow grasslands, whereas mountain meadow steppe is least affected. (3) In the future, the total area of highly suitable ragweed habitats will decrease, with a significant decrease in the area of core suitable zones (mountain steppe). Under most future scenarios and periods, the distribution centroid is projected to exhibit predominantly westward and northwestward passive displacement (maximum displacement: 0.829 km under the SSP585 scenario in the 2050s), driven by the contraction of suitable habitat in low-elevation river valleys rather than by active colonization of high-elevation zones. High-elevation alpine grassland ecosystems (mountain meadow steppe and mountain desert steppe) consistently demonstrated strong resilience against ragweed invasion across all projected periods and scenarios.

**Discussion:**

This study reveals the response pattern of ragweed, characterized by "contraction in low-elevation core areas, passive westward–northwestward displacement of the distribution centroid, and maintenance of the alpine barrier." A zoned control strategy focused on implementing physical eradication and replacement restoration in river valley core areas and establishing early warning systems in the transitional zones between mountain grasslands and meadows is recommended to safeguard regional ecological security.

## 1 Introduction

The ecological imbalance caused by biological invasions has been designated as an important and escalating danger to sustainable development worldwide. Invasive alien plants are among the key factors driving the sharp decline in biodiversity, the disruption of ecosystem services through multiple pathways, and the profound alteration of socioeconomic patterns (Rai and Singh, 2020). Moreover, damage to human health from accidents, allergies, or spread of disease can have disastrous repercussions and influence people’s ability to support themselves (Bayliss et al., 2017). The development of sustainable solutions for the preservation of ecosystems and biodiversity requires knowledge of how to forecast the spread patterns of various invasive plant species in the context of potential climate change. China has many invasive species, which severely reduce its biodiversity and harm the country’s economy (Ren et al., 2025). An evaluation of the financial costs of invasive plants worldwide revealed that from 1970 to 2017, these plants caused a conservative economic loss of $194.365 billion, averaging $4.049 billion annually. During a decade in the early 21st century, the economic costs associated with invasive plants grew at the fastest rate ever, with a year-over-year increase of 696.65%. Among the 64 countries and regions with recorded economic costs associated with invasive vegetation globally, the United States incurred the highest cumulative costs, totaling $113.913 billion. China ranked third, with total expenditures amounting to $20.831 billion (Zhao et al., 2024).

Invasive species cover a wide spectrum of adaptabilities and reproductive capacities, making them extremely difficult to eradicate once they establish themselves (Sun et al., 2024). The first step in managing foreign invasive species is to forecast their possible spread locations (Thomas and Moloney, 2015).Species distribution models (SDMs) make use of available species distribution point data combined with relevant environmental variables. Through specific algorithms, these models establish relationships between species distributions and environmental variables, enabling predictions of potentially suitable habitats for invasive species (Guisan and Zimmermann, 2000). The maximum entropy (MaxEnt) model is distinguished from other SDMs by high predictive accuracy and user-friendly software (Phillips et al., 2006). Through entropy maximization, MaxEnt estimates the likelihood of a species’ occurrence under various environmental conditions using environmental factors and current species occurrence data as inputs (Merow et al., 2013) considering a range of environmental factors, resulting in the production of precise forecasts despite having limited data (Wisz et al., 2010). MaxEnt models are widely used to predict the current and future distributions of invasive alien species, such as Senecio vulgaris L (Zhang et al., 2025), Ambrosia spp (Ki et al., 2022), Ageratina adenophora (Zhang et al., 2022), and Bidens pilosa (Chen et al., 2025); the projections for the possible distribution regions of these species are generally accurate. Additionally, studies have shown that parameter tuning greatly improves the reliability and accuracy of a model’s predictions (Vignali et al., 2020).

R, as an open-source software, has become one of the most popular statistical tools because of its easy-to-use and rich functionality and support for object-oriented programming. As of October 28, 2022, the number of packages listed in its official repository, CRAN, had reached 18,761, with this figure continuing to grow. This reflects the vibrant community ecosystem and extensive application scenarios of the software. In the field of biodiversity modeling, numerous R packages similarly support the construction of species niche models and related pre- and postprocessing workflows, providing crucial technical support for this research direction (Sillero et al., 2023). In 2019, Kuenm was published (Cobos et al., 2019). It is a software that chooses the best settings for specific studies to calibrate MaxEnt models. As a result, users can choose the best-performing MaxEnt model by running several models under various conditions. The root mean square (RMS) and feature class (FCs) were optimized using R version 4.4.1’s “kuenm” package (Cobos et al., 2019). For example, for each increment of 0.1 units, a RMS test was conducted to evaluate 31 FCS parameters, encompassing the following: hinge (H), threshold (T), product (P), linear (L), quadratic (Q), and their combinations. There were 1,240 models built in all. Based on the smallest incremental Akaike information criterion (AICc) value, statistical significance, and a false negative rate of ≤5%, the best model was chosen among those with an increment ≤2. The Kuenm R package integrates model calibration, ultimate model building, assessment, and risk analysis for extrapolation on the basis of the MaxEnt algorithm, effectively streamlining the ecological niche modeling process (Cobos et al., 2019). The functionalities provided by this tool are not yet available in other current R packages.

The perennial herb *Ambrosia artemisiifolia* L., which is a member of the Asteraceae family, is a serious invasive concern on a global scale (Hufnagel et al., 2015). Ragweed poses severe threats to biodiversity, agricultural and livestock production, ecosystems, and the environment surrounding infested areas (Ma, 2024). Ambrosia causes annual losses of more than 1 billion yuan in the agriculture, forestry, animal husbandry, and fisheries sectors (Zhou et al., 2021), and ragweed pollen and trichomes contain allergens that readily trigger allergic reactions in humans, leading to symptoms such as asthma and allergic rhinitis (Gentili et al., 2021). In approximately 2-3% of the Chinese population, ragweed pollen triggers allergies, resulting in annual medical costs of 1.45 billion yuan (Zhou et al., 2021). In 2010, ragweed and three-lobed ragweed were first discovered in Xinyuan County within the Ili River Valley region. By 2014, ragweed and three-lobed ragweed had proliferated extensively across the Ili River Valley, resulting in enhanced population dispersal capabilities and accelerated spread rates. By 2016, the distribution areas of ragweed and three-lobed ragweed had reached 1,000 and more than 200 hm^2^, respectively (Wen et al., 2020). By 2019, the distribution areas of ragweed (*A. artemisiifolia*) and giant ragweed (*A. trifida*) across the entire Ili River Valley had reached 136,200 and 37,900 hm^2^, respectively, and the ranges of both species continue to expand (Dong et al., 2020). While ragweed colonizes predominantly roadsides, farmland margins, and riverbanks, giant ragweed has been documented not only in agricultural habitats but also in native grassland areas, posing a serious threat to grassland ecosystems (Dong et al., 2020) These conditions have severely affected local agriculture and animal husbandry. Moreover, owing to the strong regenerative capacity and ability of ragweed and trifoliate ragweed to survive underground for up to 40 years (Song et al., 2012), local governments are facing difficulties implementing prevention and control measures.

In this study, various shared socioeconomic pathway (SSP) scenarios were used to systematically model the spatiotemporal evolution and possible suitable habitat of ragweed (*A. artemisiifolia*) under present conditions and across future periods from the 2030s to the 2090s. A parameter-optimized MaxEnt model based on field-collected geographic distribution data for ragweed in Xinyuan County was utilized. This research revealed important environmental drivers that constrain the distribution of ragweed, and a detailed assessment of its invasion risk and spatial patterns at the county scale was conducted. Through parameter optimization adjustments, the accuracy of the prediction results was enhanced significantly. This study provides a scientific foundation for sustainable ecosystem management in Xinyuan County and the development of accurate control strategies by combining data from local sampling with multiple climate scenarios to assess the ecological adaptability and possible expansion trends of ragweed under future climate change.

## 2 Materials and methods

### 2.1 Research area

Xinyuan County is located in the western part of the Xinjiang Uygur Autonomous Region and is nestled in the eastern Ili River Valley deep within the Tianshan Mountains. Its geographical position is highly strategic, spanning 82°28′ to 84°57′ east longitude and 43°01′ to 43°40′ north latitude, with a total area of 7,580.62 km^2^. The county boasts a well-developed transportation network, with National Highways 217 and 218 intersecting Provincial Highway 316, making it a vital hub connecting Central Asia, northern Xinjiang, and southern Xinjiang. Xinyuan County features distinct topography, surrounded by mountains on three sides, while the west opens into a relatively broad plain, presenting an overall natural terrain gradient that slopes from east to west.

**Figure 1:**
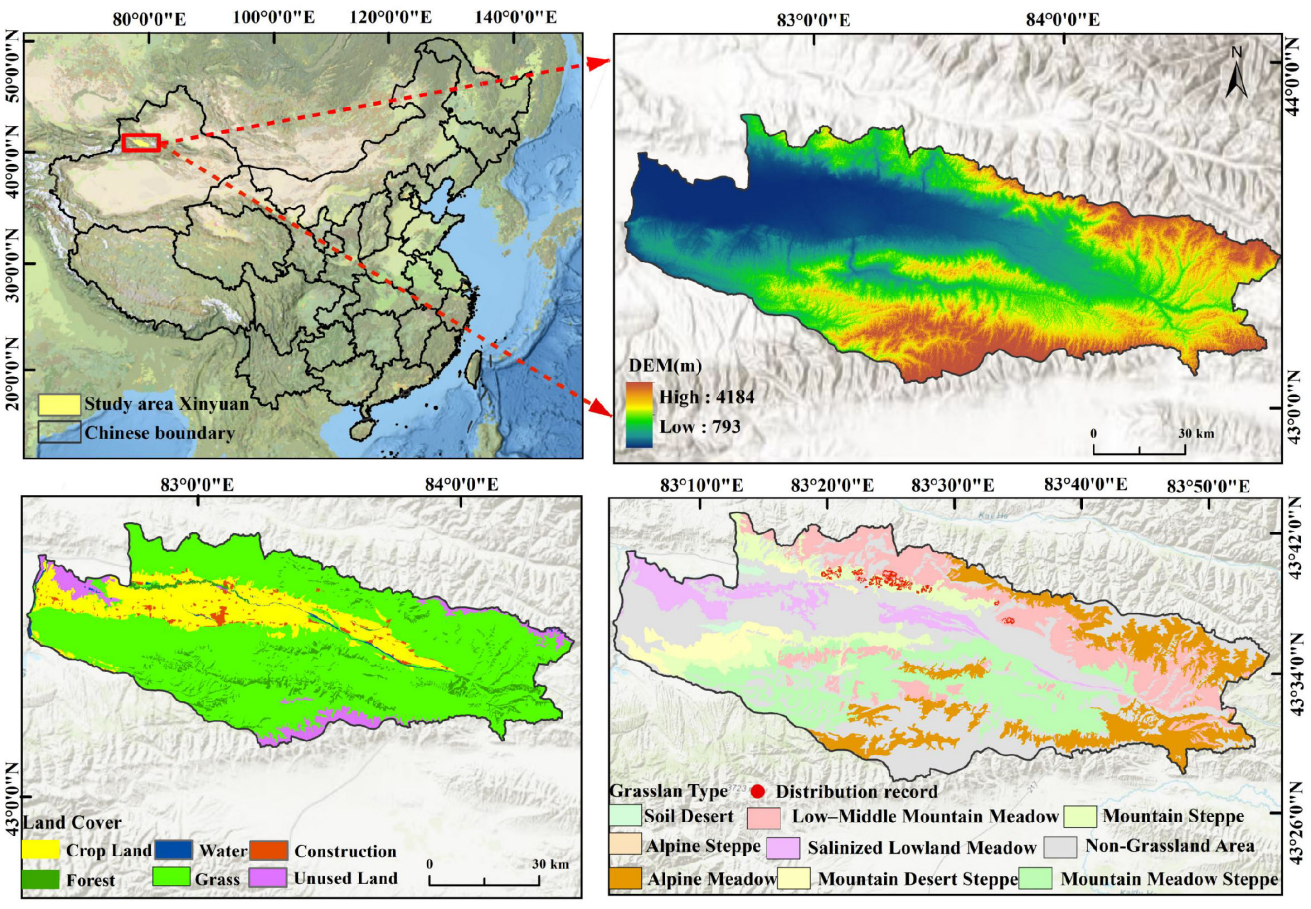
Map of the study area.

The county features undulating mountains, with mountainous terrain covering 73.2% of its total area, suggesting that it has distinct mountainous characteristics (Yuan et al., 2025). Xinyuan County is a major outbreak area for ragweed and trifoliate ragweed and is situated in the eastern Ili River Valley, in the center of the Gongnaisi Grassland. The county has a moderate continental semiarid climate, with an average annual temperature of 8.1 °C and 480 mm of precipitation (**Figure 1**).

### 2.2 Data collection

From 2019 to 2025, during the ragweed growing season (May–June), surveys and measurements were conducted each year in the distribution areas of ragweed species within Xinyuan County. More than 2,000 sample points were collected. Distribution points were screened using ENMTools software to retain only one distribution point per five grid cells. After further refinement, 300 valid distribution points were ultimately utilized as model inputs. These records were subsequently randomly partitioned into a training dataset (75%, n = 225) and an independent test dataset (25%, n = 75) for model calibration and evaluation, respectively.

### 2.3 Environmental variables

**Figure 2:**
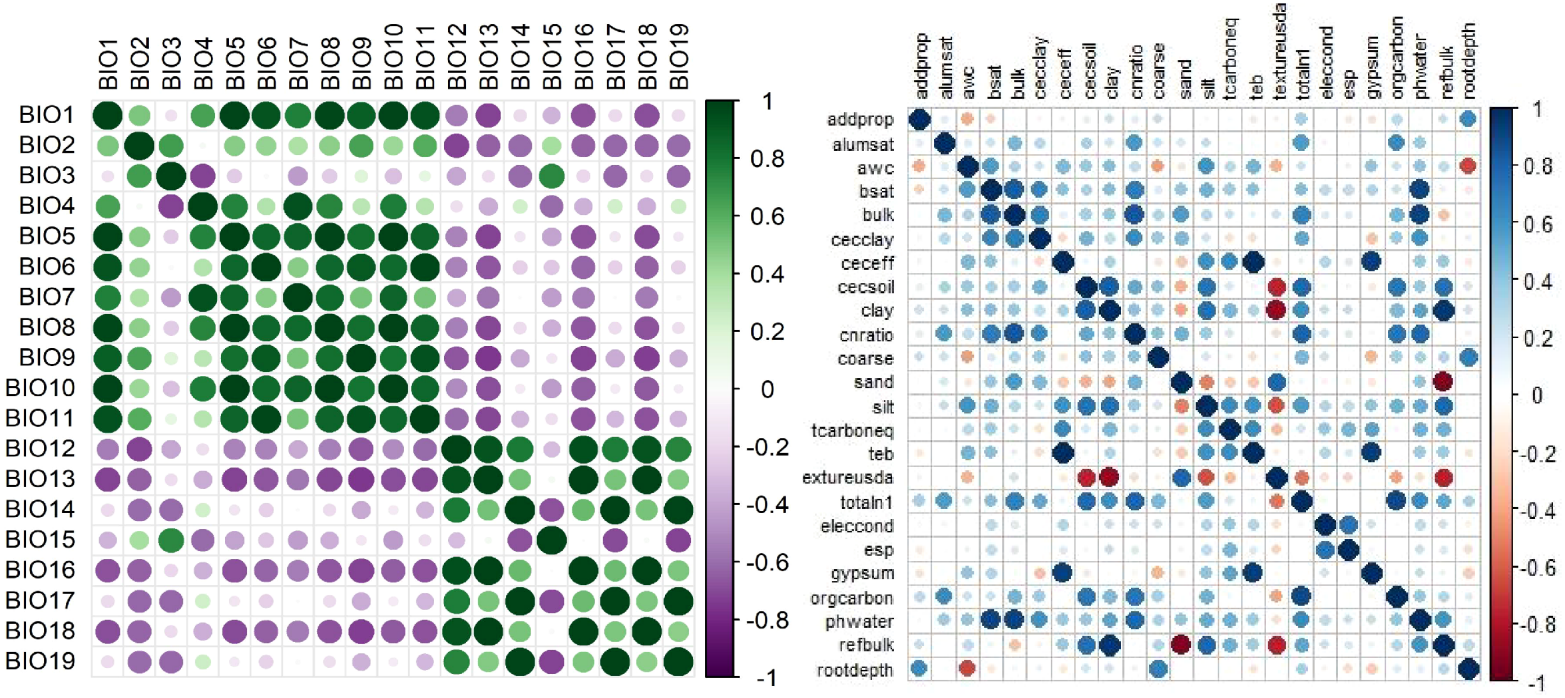
Correlation matrix of 19 bioclimatic variables and 24 soil variables.

Bioclimatic variables for 19 distinct periods and three topographic variables at 2.5’ (5 km × 5 km) resolution were acquired from https://www.worldclim.org. These periods are as follows: present (1970–2000 average), 2030s (2021–2040 average), 2050s (2041–2060 average), 2070s (2061–2080 average), and 2090s (2081–2100 average). Based on the CMIP6 SSP-representative concentration pathway (RCP) scenario combinations, future bioclimatic variables were predicted using the Beijing Climate Center climate system model with a 2-m resolution (BCC-CSM2-MR). In this study, SSP126, SSP245, SSP370, and SSP585 represent low-, medium-, high-, and very high-emission scenarios, respectively (Su et al., 2021). Furthermore, 24 soil variables at a precision of 30 arcseconds (1 km × 1 km) were obtained from the FAO Harmonized World Soil Database (HWSD; https://gaez.fao.org/pages/hwsd), where the prefix “D*_” in variable names denotes the data layer following the HWSD naming convention. ENM tools were used to determine connections between bioclimatic and soil variables. Environmental factors with high correlations (|r| > 0.8) were removed, yielding a final selection of 22 variables (see **Table 1**). The correlation coefficient matrix is shown in **Figure 2**.

**Table 1:** Variables utilized in model prediction.

Variable description	Abbreviated name	Unit	Data sources
Annual mean temperature	BIO1	(°C)	WorldClim version 2.1 (https://www.worldclim.org/)
Isothermality	BIO3	%	
Temperature seasonality	BIO4	(°C)	
Temperature annual range	BIO7	(°C)	
Annual precipitation	BIO12	mm	
Precipitation in the driest month	BIO14	mm	
Precipitation seasonality	BIO15	%	
Precipitation in the driest quarter	BIO17	mm	
The available water storage capacity of the soil	D*_AWC	mm	https://gaez.fao.org/pages/hwsd
Soil base saturation	D*_BSAT	%	
The cation exchange capacity of the soil	D*_CEC_SOIL	cmol/kg	
The cation exchange capacity of clayey soil	D*_CEC_CLAY	cmol/kg	
The fineness of soil sand grains	D*_COARSE	% vol	
Exchangeable sodium salts	D*_ESP	%	
The depth of soil that can be rooted	D*_ROOT_DEPTH	cm	
Sludge content	D*_SILT	% wt	
USDA soil texture classification	D*_TEXTURE_USDA		
China Land Cover Dataset	CLD	-	
Aspect	ASPECT	-	Derived from Elev
Elevation	DEM	m	Geospatial data cloud platform (https://www.gscloud.cn/)
Slope	SLOPE	(°)	Derived from Elev
Grazing intensity	GRAZING INTENSITY		https://www.resdc.cn/

The prefix “D*_” denotes soil variables derived from the FAO Harmonized World Soil Database (HWSD; https://gaez.fao.org/pages/hwsd), where “D” represents the data layer.

### 2.4 MaxEnt model and the kuenm package

The MaxEnt model uses existing data to forecast the suitable distribution ranges of species while accounting for present and future climate conditions. It does this by combining statistical modeling concepts with effective machine learning methods. MaxEnt has been widely applied. By combining the actual distribution samples of the target species with environmental data reflecting current climate conditions, areas suitable for the target species within the predicted region can be identified through specific algorithms and model construction (Bock et al., 2016). All the MaxEnt analyses were conducted using MaxEnt version 3.4.4 (Phillips et al., 2006), implemented through the kuenm package in R 4.4.1 (Cobos et al., 2019). A total of 10,000 background points were randomly generated within the study area (Xinyuan County) to characterize the environmental conditions available across the landscape. Prior to spatially simulating the current and future distributions of invasive ragweed (*A. artemisiifolia*), the MaxEnt model was optimized by testing various combinations of model parameters to increase prediction precision and resilience. The parameter tuning process was implemented using the kuenm package within the R programming environment. This tool was used to systematically evaluate multiple configurations of FCs and regularization multipliers (RMs) to identify optimal modeling parameters. As a highly optimized modeling support tool, the kuenm package has gained widespread recognition among researchers for its reliability and efficiency in parameter selection in species distribution modeling studies (Li et al., 2024; Zhao et al., 2024). We evaluated 31 FC combinations, comprising all possible combinations of L, Q, P, T, and H feature types, alongside 40 RM values ranging from 0.1 to 4.0 in increments of 0.1, yielding a total of 1,240 candidate parameter combinations. Models with statistically significant performance and omission rates ≤ 5% were retained as candidate models (Cobos et al., 2019).

The suggested model for ragweed was determined to be the one with a delta AICc < 2 (Warren and Seifert, 2011). From these candidates, the model with the lowest delta AICc value was identified as the final optimal model, which was configured with an RM of 3.2 and an FC of linear, quadratic, and threshold (LQT). The filtered distribution point and environmental data were input into the MaxEnt model. The jackknife, random seed, and ‘generate response curve’ approaches were chosen to assess the relative contributions of the environmental factors under default settings. Ten iterations of the procedure were implemented using the optimal settings for the FC and RM.

The ranges for interpreting training area under the curve (AUC) values were 0.5–0.6 (unacceptable), 0.6–0.7 (bad), 0.7–0.8 (fair), 0.8–0.9 (good), and 0.9–1.0 (outstanding) (Zhao et al., 2021).

### 2.5 Habitat suitability classification

According to the estimated species distribution map, there is a 0–1 chance that a given habitat is suitable. A high probability that the species will exist in a certain area is indicated by values near 1 (Bai et al., 2022). We utilized five classifications to identify appropriate habitats: unsuitable (< 0.10), low suitability (0.10–0.28), moderate appropriateness (0.28–0.48), high suitability (0.48–0.69), and extremely suitable (>0.69). The specific threshold values were determined by applying the Jenks natural breaks method in ArcGIS 10.8 to the predicted suitability raster of the present-day model output, following the five-class classification framework adopted in previous studies (Hosni et al., 2022).

## 3 Results

### 3.1 Assessment of model performance and contributions of environmental variables to the model

**Figure 3:**
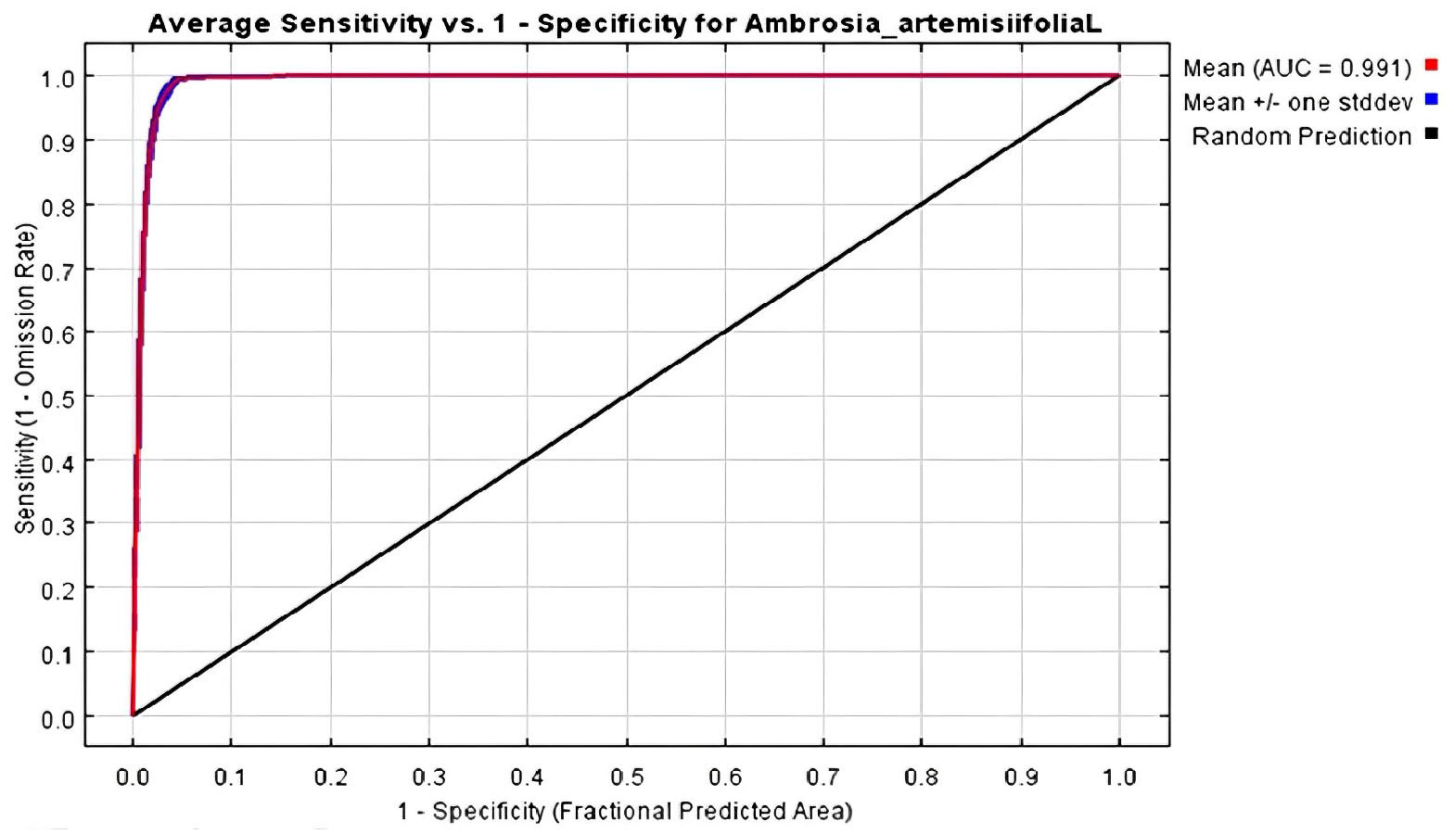
ROC analysis of the MaxEnt results.

The potential distribution of ragweed (*A. artemisiifolia* L.) was simulated using the MaxEnt model with 10 replicated runs. Parameter optimization via the kuenm package yielded an optimal model configured with a FC of and a RM of 3.2. Although the model achieved a high average training AUC of 0.991, independent metrics were prioritized to preclude potential overestimation of the predictive capacity caused by overfitting. Consequently, the optimized model demonstrated robust performance, achieving a partial receiver operating characteristic (ROC) value of 1.959 (*p* < 0.001) and an empirical omission value of 0.0267 at the 5% threshold. These comprehensive evaluations confirmed that the model accurately captured the environmental drivers of the ragweed distribution, providing credible and nonoverfitted spatial projections (**Figure 3**).

Key environmental factors affecting the spread of ragweed in Xinyuan County were identified in this study using the contribution values and replacement importance matrix obtained with the MaxEnt model (**Table 2**). The findings of the analysis reveal that hydrothermal seasonal patterns dominate the relative contributions of environmental parameters. Specifically, precipitation seasonality (BIO15), isothermality (BIO3), temperature seasonality (BIO4), and annual precipitation (BIO12) constitute the primary explanatory variable set, constituting 68.3% of the total contribution to the modeling results.

**Table 2:** Contributions of the top ten environmental factors and their cumulative proportions.

Variable	Contribution Percentage	Permutation Importance
Precipitation seasonality BIO15	23.1	9.7
Isothermality BIO3	21.4	10.2
Temperature seasonality BIO4	14.3	6.4
Annual precipitation BIO12	9.5	12.6
SLOPE	6.1	17.1
ASPECT	4.7	4.9
Elevation	4.3	4.8
GRAZING INTENSITY	4.3	4.8
BIO7	2.9	8
Rootdepth	1.4	14.3

Among these, BIO15 (23.1%) and BIO3 (21.4%) emerged as the most critical predictors, reflecting the significant constraints imposed by an uneven precipitation distribution and diurnal temperature variations on ragweed invasion and spread. Replacement importance analysis revealed the potential ecological weights of nonclimatic factors: slope (SLOPE) ranked first in terms of replacement importance (17.1%), in contrast to its relatively low contribution (6.1%), and BIO12 also displayed higher replacement importance (12.6%) than its contribution rate (9.5%). This asynchrony between contributions and replacement importance suggests that terrain heterogeneity and total water availability provide crucial additional information gains for the model, serving as core parameters ensuring the robustness of predictive outcomes.

### 3.2 Relationships between environmental variables and ragweed occurrence probability

**Figure 4:**
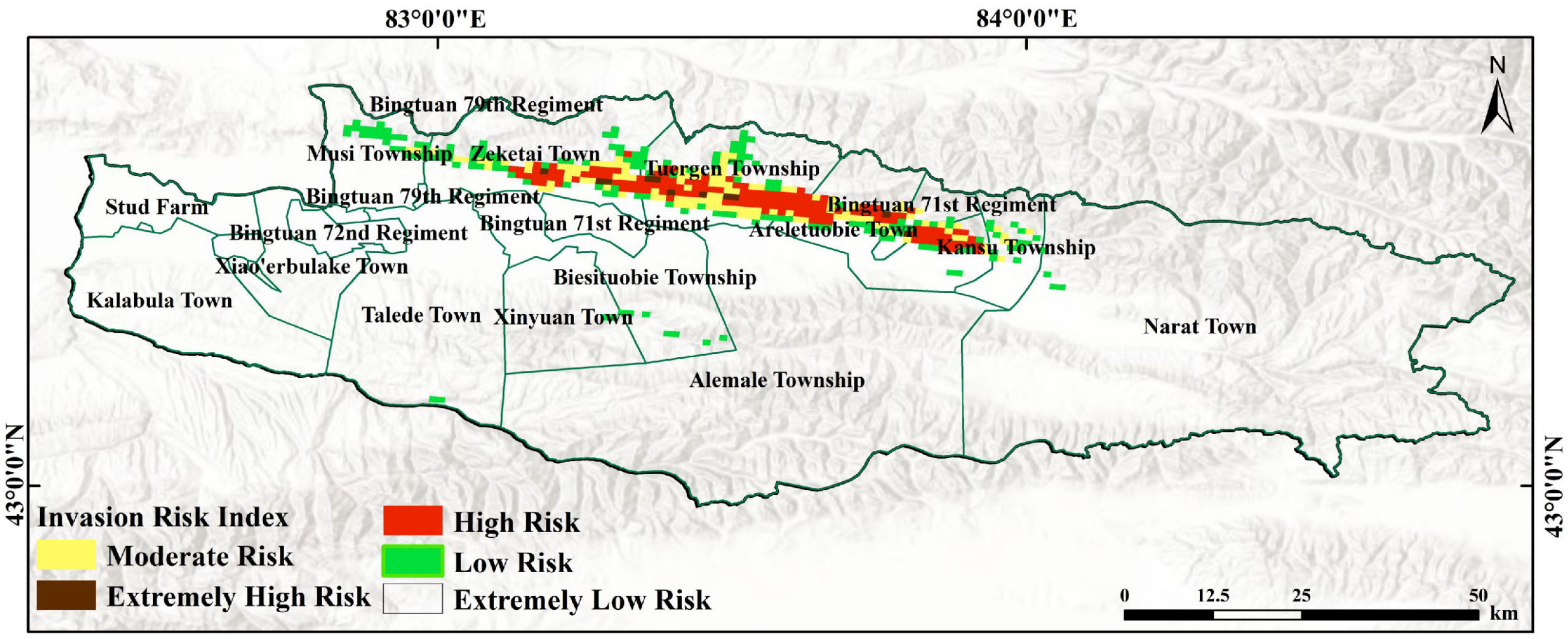
*Ambrosia artemisiifolia* L. areas in Xinyuan given the existing climate. The range of the appropriateness index is 0.0 (poor suitability) to 1.0 (high suitability). The ragweed distribution was classified into five grades using the natural breaks suitability classification method.

**Figure 5:**
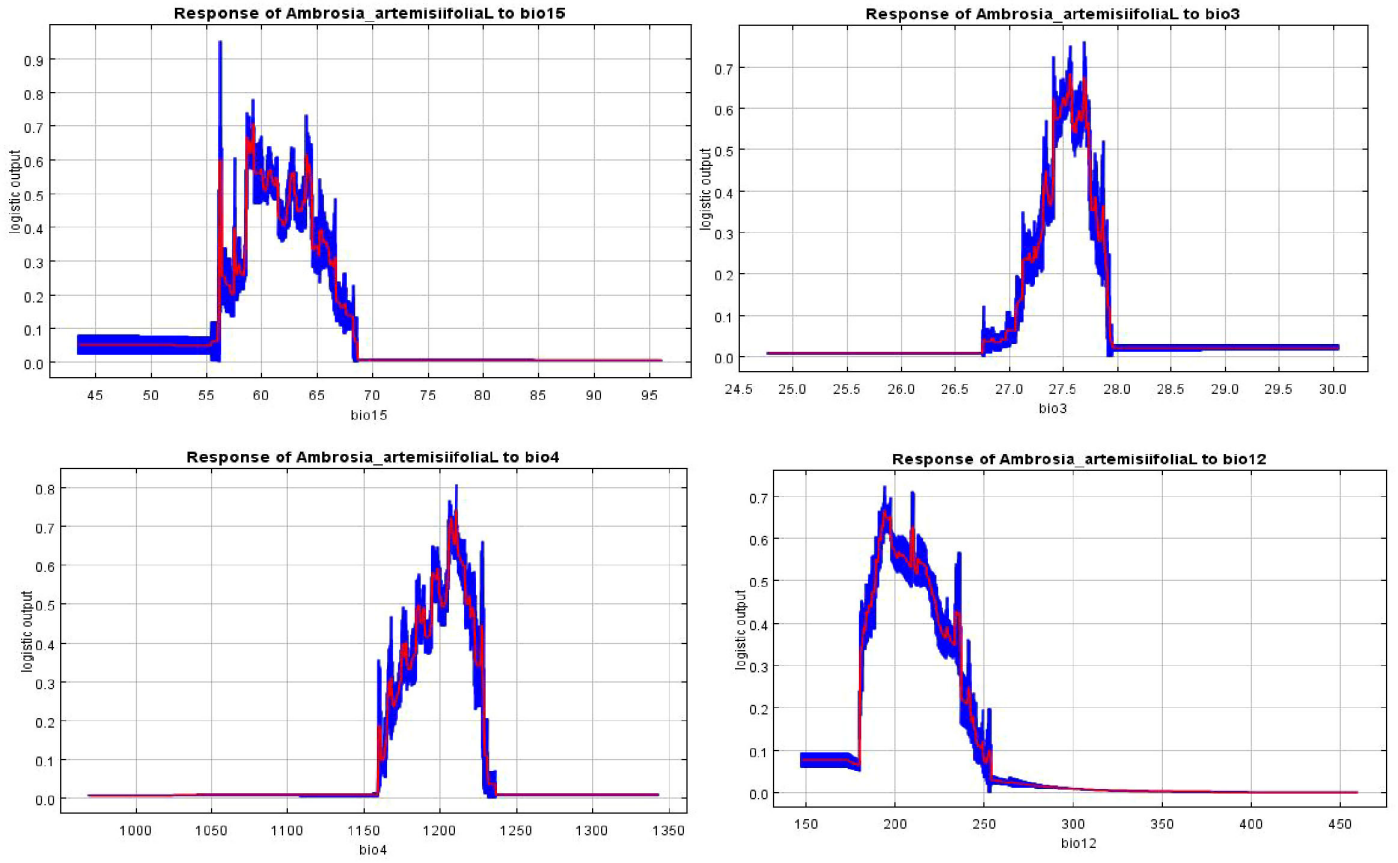
Response curves of important environmental factors in models of the ragweed distribution.

The response curves of the four main environmental factors (BIO15, BIO3, BIO4, and BIO12) revealed the specific ecological niche characteristics of ragweed in Xinyuan County (**Figure 4**). Among these, BIO15 exhibited a steep nonlinear response, peaking at approximately 59%, with a broad suitable range (57%-66%), indicating that the species prefers habitats with moderate seasonal precipitation fluctuations. BIO3 exhibited an extremely narrow unimodal pattern, with the suitable range strictly confined between 27.2% and 27.9% (the peak at 27.6%-27.8%), reflecting its high sensitivity to temperature stability (**Figure 5**).

The BIO4 displayed a bell-shaped distribution, with a peak at 1210–1220 and a suitable range of 1190–1230, indicating that excessive thermal variations limit the colonization of the species; the BIO12 had distinct left-skewed characteristics and a threshold effect, with the optimal range concentrated from 185–240 mm (peak at 190–200 mm). The low BIO12 optimum (185–240 mm) is consistent with the spatial concentration of occurrence records in low-elevation river valley alluvial plains (Section 3.3). In the Tianshan Mountain system, BIO12increases characteristically with elevation; consequently, valley-floor grid cells exhibit BIO12 values substantially below the county-wide mean of approximately 480 mm. The modeled precipitation optimum therefore primarily reflects the hydrological conditions of the valley-floor habitats where ragweed is predominantly established. Additionally, river valley alluvial plains naturally receive supplemental water inputs from lateral river channel flow and surface runoff, further supporting the establishment of ragweed in these settings.

### 3.3 Habitat suitability of ragweed in Xinyuan County during the current time period under optimal parameters

Based on the simulation results of the optimal parameters predicted with the MaxEnt model, notable spatial heterogeneity and a clustered pattern were detected along the river valleys in Xinyuan County’s possibly suitable ragweed (*A. artemisiifolia*) environment (**Figure 4**). Although the total area of risk zones is 327 km^2^, accounting for only 4.32% of the county’s total area, the potential threat to ecological security cannot be overlooked. High-risk and extremely high-risk zones constitute the core areas suitable for ragweed invasion, covering a combined area of 81 km^2^. These zones spatially align with low-elevation river valley alluvial plains, concentrated in Tuergen Township, Zeketai town, Aretuobai town, Kansu town, and portions of Corps 71 and Corps 79. These areas typically feature favorable water and heat conditions alongside frequent human activity, making them high-risk zones for ragweed colonization and outbreaks. The moderate-risk zone (108 km^2^) and low-risk zone (138 km^2^) primarily surround these core areas as peripheral buffer belts, extending westward to cover Mus Township. This pattern indicates that the species tends to spread primarily along river valley plains and transportation corridors. Moreover, 95.68% of the county’s territory (low-risk zones) consists of high-altitude mountainous areas in the south. The cold climate in these regions not only makes them unsuitable for ragweed growth but also forms a natural geographical barrier, limiting the further southward expansion of the species.

**Figure 6:**
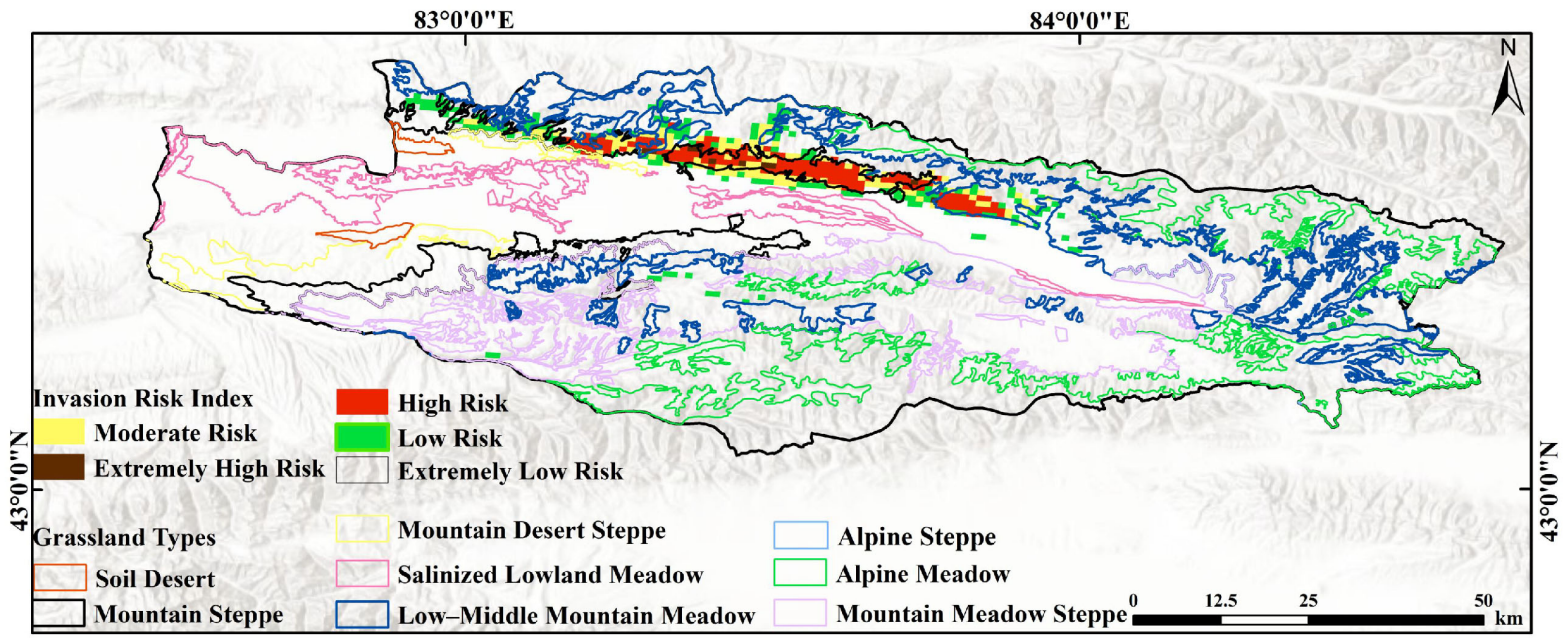
Distribution characteristics of potentially suitable habitats for *Ambrosia artemisiifolia* across different grassland types.

To further clarify the grassland ecosystem types most threatened by ragweed, we spatially overlaid the invasion risk map with the grassland classification map of Xinyuan County (**Figure 6**). Quantitative analysis revealed significant spatial heterogeneity and habitat selectivity in the distribution of potentially suitable habitats for ragweed across grassland types. Specifically, the mountain steppe was identified as the habitat most vulnerable to ragweed invasion. This grassland type exhibited the greatest spatial coupling with high-suitability zones, encompassing 73 km^2^ of high-risk areas and 6 km^2^ of extremely high-risk areas. Together, these factors dominated the core threat zone, indicating that the hydrotemperate conditions of this habitat are highly conducive to ragweed colonization and population outbreaks. Low–middle mountain meadows exhibit secondary susceptibility. Although this land cover type includes extensive low-risk areas (42 km^2^) and medium-risk areas (30 km^2^), it still contains 38 km^2^ of high-risk zones and 2 km^2^ of extremely high-risk zones. These findings indicate that while low-to-middle mountain meadows exhibit slightly greater overall resistance than mountain meadow steppes do, they remain a significant potential corridor for the expansion of ragweed to higher elevations and warrant intensive monitoring. In contrast, other grassland types exhibit stronger environmental resistance. The mountain meadow steppe contained only 9 km^2^ of low-risk areas, with no medium-risk or high-risk zones detected, indicating that this habitat significantly suppressed ragweed growth. Mountain desert steppes also face extremely low threat levels, with both at-risk and high-risk areas covering only 1 km^2^. Ambrosia control strategies in Xinyuan County should focus on these two core habitats: mountain steppe and low-to-mid-elevation mountain meadows.

### 3.4 Future distribution of ragweed in Xinyuan County under optimal parameters

**Figure 7:**
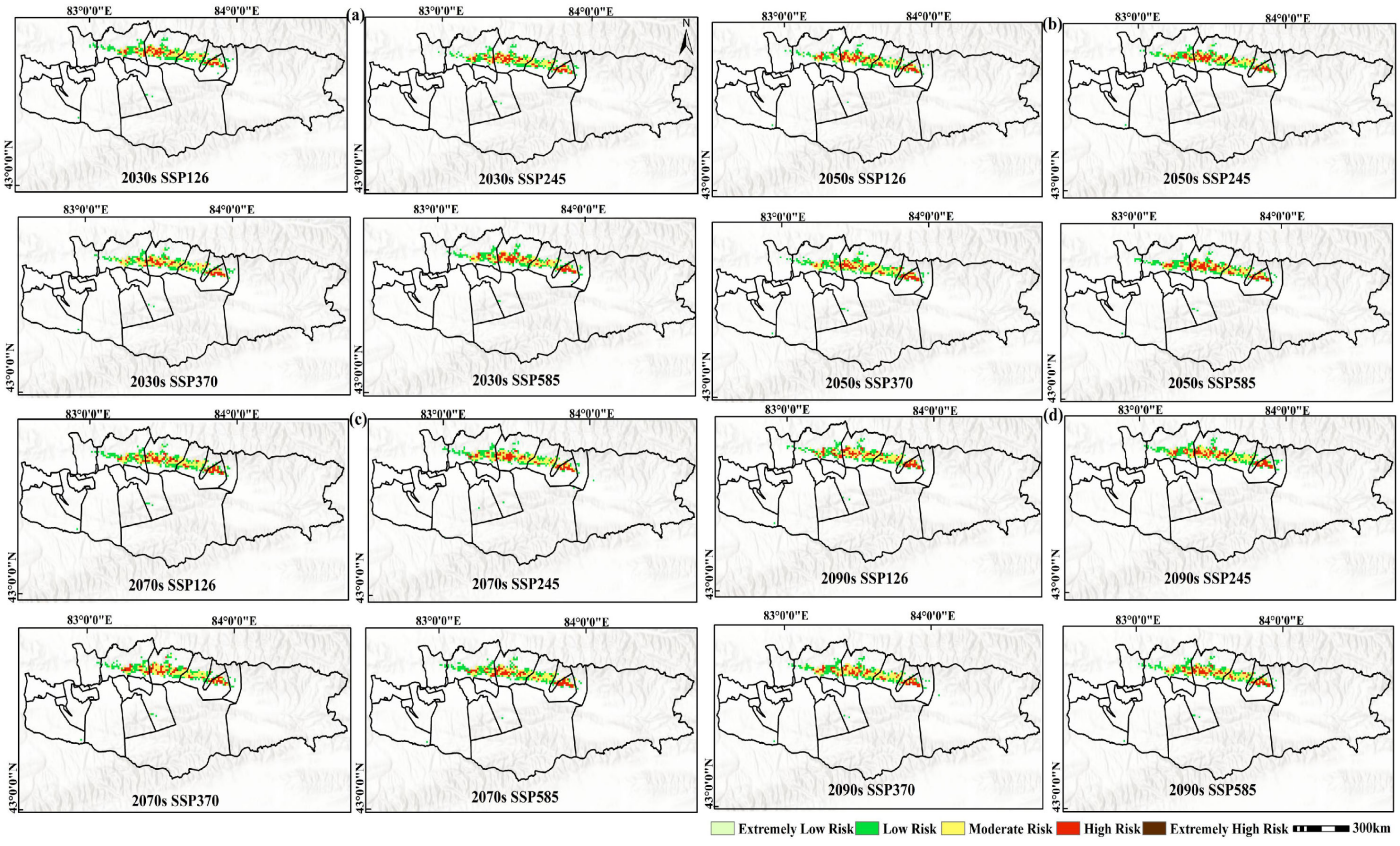
Dynamic modifications to the distribution of ragweed invasion risk under future climate scenarios: **(a)** 2030s, **(b)** 2050s, **(c)** 2070s and **(d)** 2090s.

**Figure 8:**
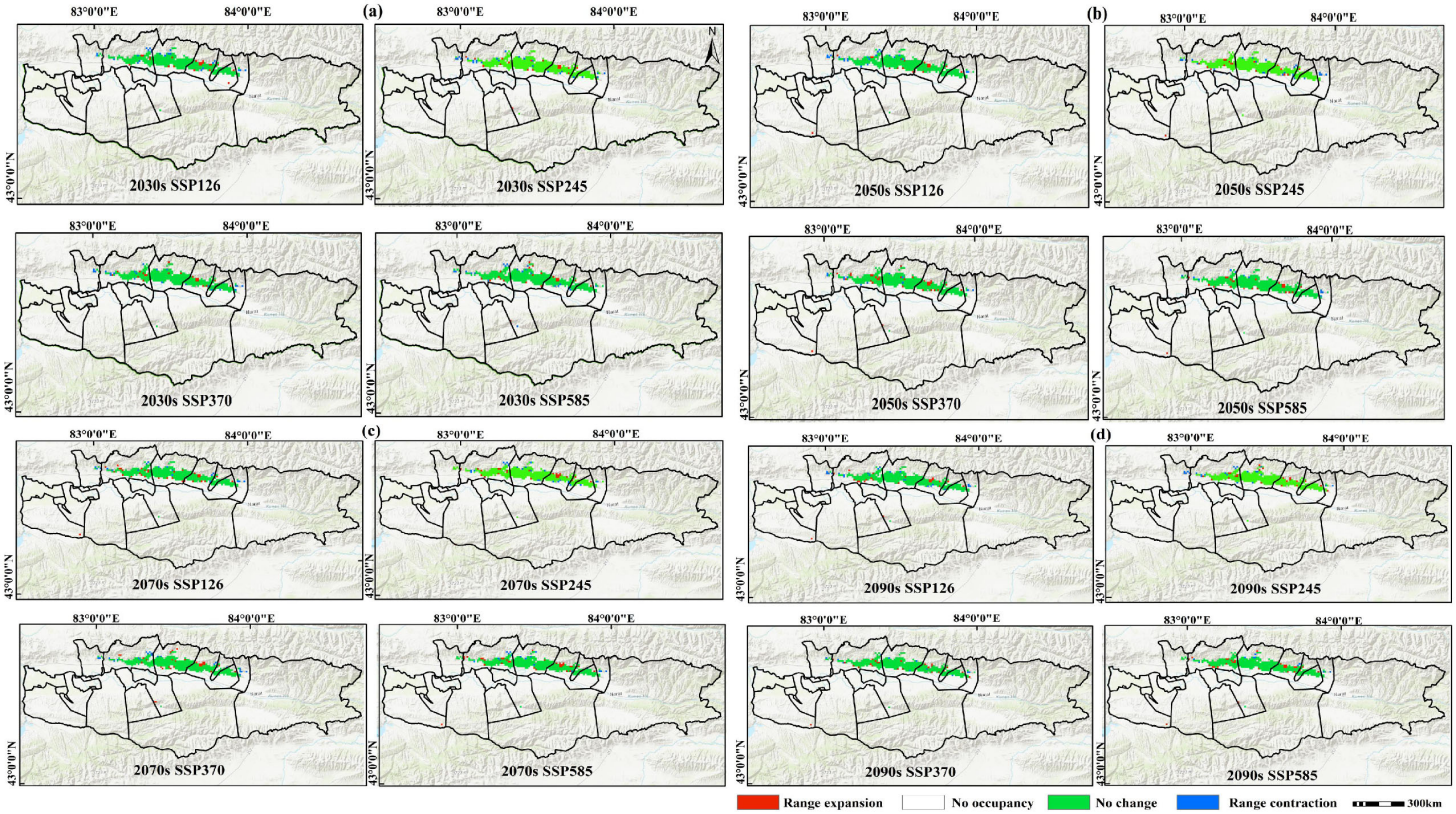
Distribution dynamics of the ragweed invasion area under future climate scenarios: **(a)** 2030, **(b)** 2050, **(c)** 2070, and **(d)** 2090.

Simulation results based on optimal model parameters indicated that under future climate change impacts, the potential invasion risk pattern of ragweed in Xinyuan County will undergo significant restructuring (**Figure 7**). Compared with the 2020 baseline conditions, the overall trends across all the periods (2030s to 2090s) under the four climate scenarios (SSP126, SSP245, SSP370, and SSP585) sharply decreased in areas classified as having high invasion risk, with reductions ranging from 38.36% to 50.68%. The greatest decline occurred in the 2030s under the SSP245 scenario, indicating the high sensitivity of this risk level to climate change. Concurrently, areas classified as medium and low risk exhibit fluctuating changes. The medium-risk areas showed variations ranging from a 10.77% decrease to a 3.08% increase, whereas the low-risk areas fluctuated between a 16.56% decrease and a 19.02% increase, reflecting the dynamic spatial shifts in invasion risk. The total area at risk across all risk levels was projected to be lower than the current level under all future scenarios, with reductions ranging from 11.62% to 26.20% (**Figure 8**). Notably, the total risk area reached its minimum under the SSP245 scenario in the 2030s, whereas it relatively rebounded in the 2090s under the SSP585 scenario, which represents a high-forcing pathway, becoming the greatest among all the scenarios. This phenomenon suggests that although the future climate conditions will generally suppress the total suitable range of ragweed and cause severe decreases in highly suitable habitats, prolonged high-intensity warming may alter environmental conditions, leading to shifts in risk levels across certain regions. Consequently, the geographic distribution of ragweed invasion hotspots may increase.

**Figure 9:**
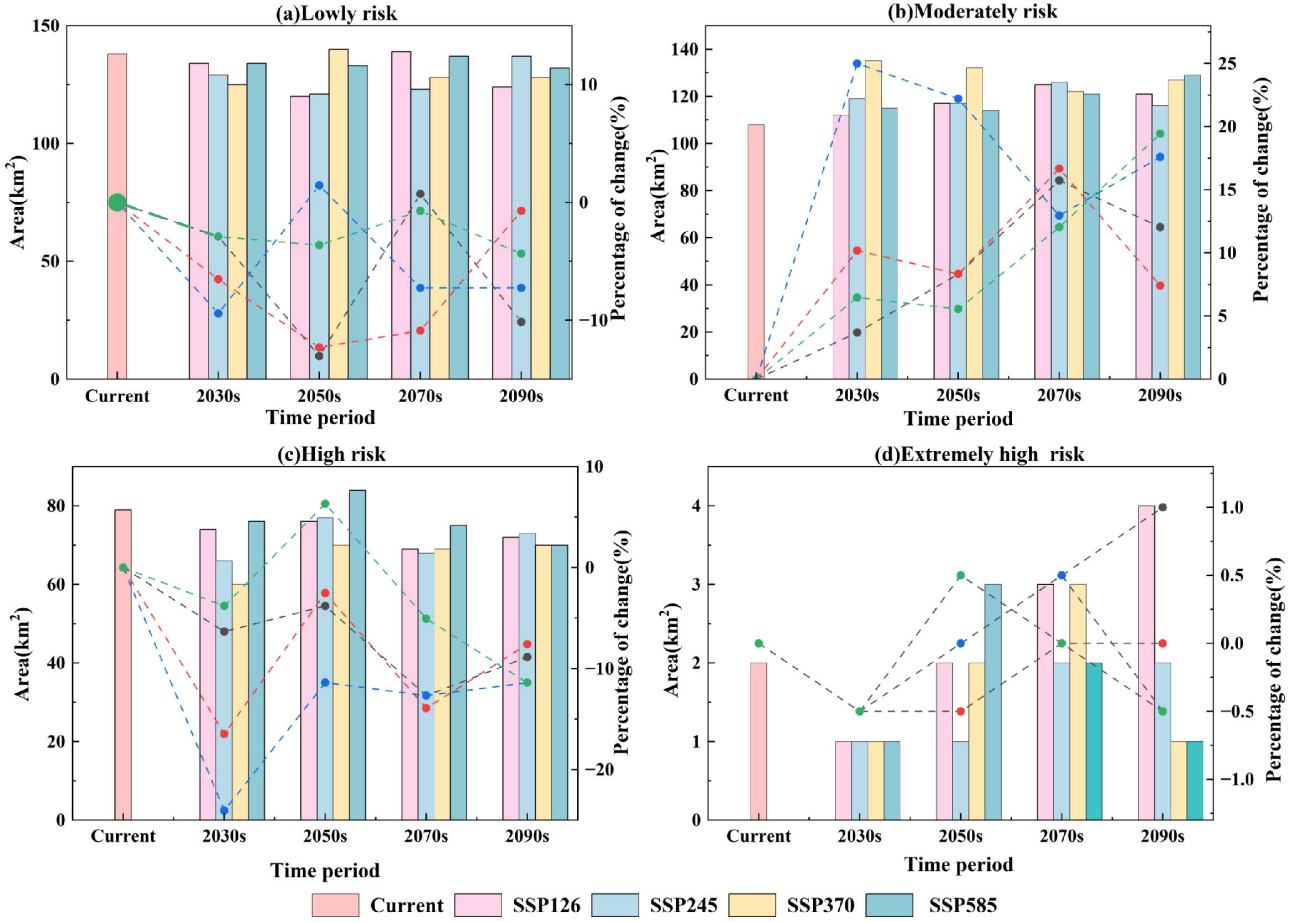
Changes in the ragweed invasion area and proportion under future climate scenarios. The bars represent area values, while the lines indicate percentage changes.

Based on the spatiotemporal dynamic simulation data map and spatial distribution map (**Figure 8**, **Figure 9**), the future distribution pattern of ragweed in Xinyuan County exhibited a composite evolutionary characteristic of “broad contraction, stable core, and localized expansion.” Spatially, the core suitable area demonstrated exceptional climatic resilience, consistently covering 306–326 km^2^ (approximately 73% of the baseline suitable area). It currently broadly occupies the alluvial plains in the central Ili River Valley and is unlikely to naturally retreat because of climatic fluctuations. In contrast, habitat contraction zones dominate the fluctuating areas, with areas varying between 105 and 126 km^2^ (approximately ten times the expansion area). These changes primarily occur in the transitional zones where the river valley margins meet the foothills. These findings indicate that while future climate change will significantly suppress species survival in marginal habitat areas (with a negative net rate of change), it cannot affect its core population pool.

However, the risk of “directional expansion” of suitable habitats is particularly critical. Although the expansion area is only 7–16 km^2^, its spatial distribution tends to penetrate higher-elevation areas or upstream tributaries of rivers. Particularly in the long-term projection (2090s) under the high-emissions SSP5-8.5 scenario, the expansion area peaks at 15 km^2^ with increasing radiative forcing and nearly doubles compared with that under the low-emissions scenario during the same period. This characteristic indicates that while extreme warming and humidification limit marginal populations, they drive the species to overcome native habitat constraints and undergo targeted migration and colonization into previously unsuitable areas. This constitutes a new frontier of invasion that must be guarded vigilantly against in future prevention and control efforts.

### 3.5 Response of invasion risks in different grassland patterns to future climate warming

**Figure 10:**
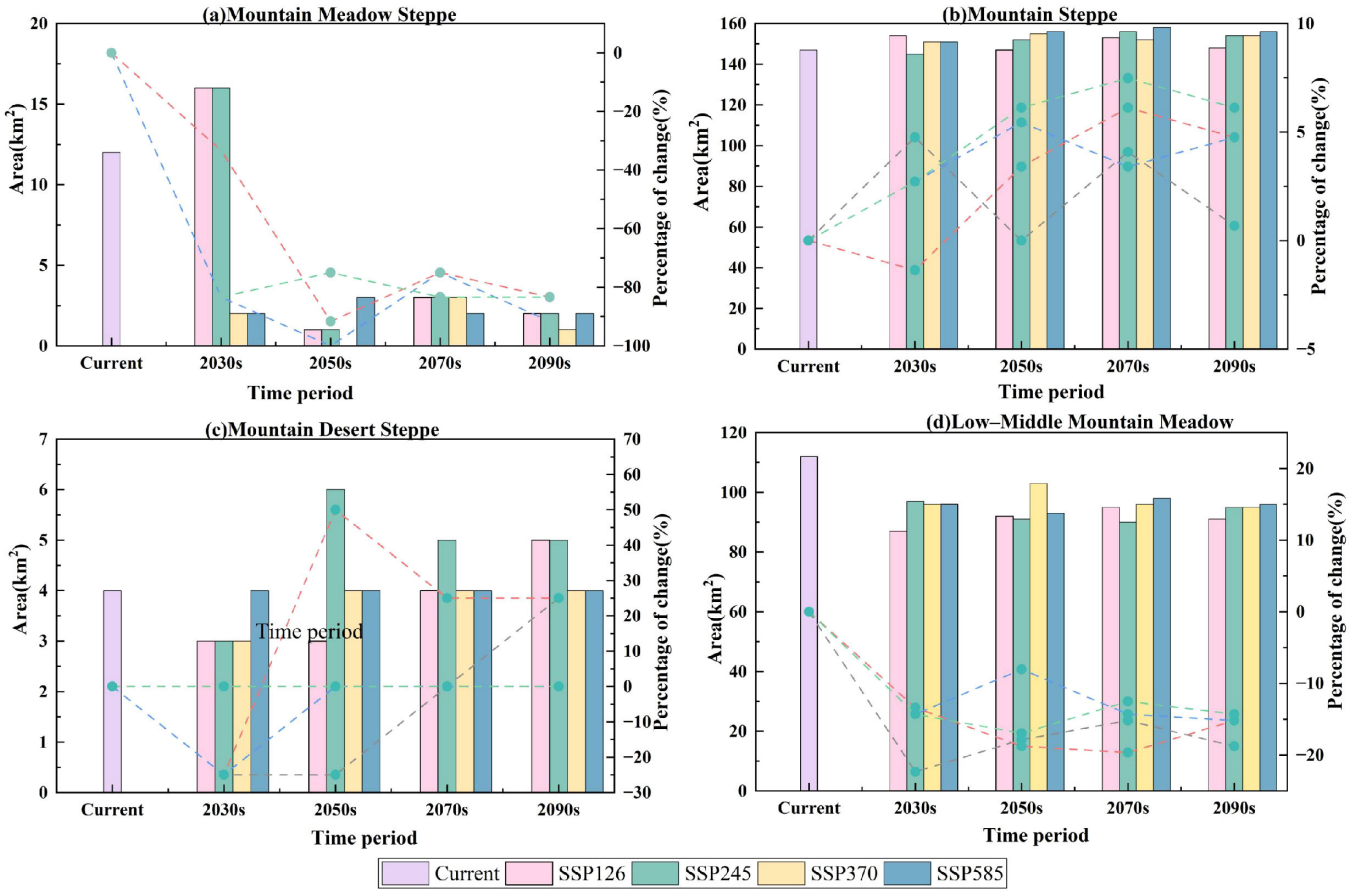
Changes in the area and percentage of ragweed invasion across different grassland types under future climate scenarios. The bars reflect the area values, whereas the lines indicate percentage changes.

Overlay analysis based on future climate scenarios revealed a significant restructuring of invasion risks within specific grassland ecosystems. Contrary to the assumption of large-scale expansion toward higher-elevation grasslands, quantitative data indicated that invasion risks within primary susceptible habitats exhibited a pronounced trend of “high suitability degradation and a downward shift in risk levels” (**Figure 10**).

Under future warming scenarios, mountain steppe, which is currently the most susceptible ecosystem, is predicted to experience a sharp reduction in habitat quality. Large portions of this habitat are currently suitable for ragweed survival, according to the data, but the intensity of the threat is predicted to change qualitatively. In most future scenarios, the area designated as high risk is expected to stabilize between 39 and 42 km^2^, a significant decrease from the current 73 km^2^ (e.g., SSP2-4.5 in the 2030s and SSP5-8.5 in the 2090s). In stark contrast, medium-risk areas are projected to exhibit compensatory growth, which is projected to surge from the current 39 km^2^ to 63–77 km^2^. This “massive shift from high to medium risk” indicates that future climatic conditions, likely due to excessive warming or moisture limitations, are diminishing the carrying capacity of mountain grasslands as “optimal habitats,” forcing the invasive core zone to transition from an “explosive state” to a “sustained state”.

The low–middle mountain meadow also showed a similar trajectory. The area of high-risk zones within this subhabitat will decrease from the current 38 km^2^ to a projected future range of 24–31 km^2^. Notably, the current 2 km^2^ of extremely high-risk areas are projected to disappear or shrink to negligible levels under most scenarios (e.g., zero invasions under the SSP2-4.5 scenario by the 2030s). These findings further confirm that future climate change will inhibit the formation of high-density invasion hotspots in low-to-mid mountain meadows.

For high-altitude ecosystems such as the mountain meadow steppe and mountain desert steppe, data indicate sustained resistance to invasion. Across all the examined periods and emission scenarios, high-risk areas for these grassland types remained at negligible levels (0–1 km^2^), with no evidence of significant upward migration or colonization. For instance, even under the high-emissions SSP5-8.5 scenario projected for the 2090s, the mountain meadow steppe retained only 2 km^2^ of low-risk zones, with no high-risk distributions recorded.

The response pattern of grasslands to climate warming manifests primarily as a structural shift in risk intensity rather than a series of spatial breakthroughs into new ecosystem types. Core invasion pressures on mountain steppes and low- to mid-elevation mountain meadows are projected to transition from a “high-intensity clustering” pattern to a “medium-intensity diffusion” pattern.

### 3.6 Shift in the centroid of the ambrosia invasion potential distribution in several future climate scenarios

**Figure 11:**
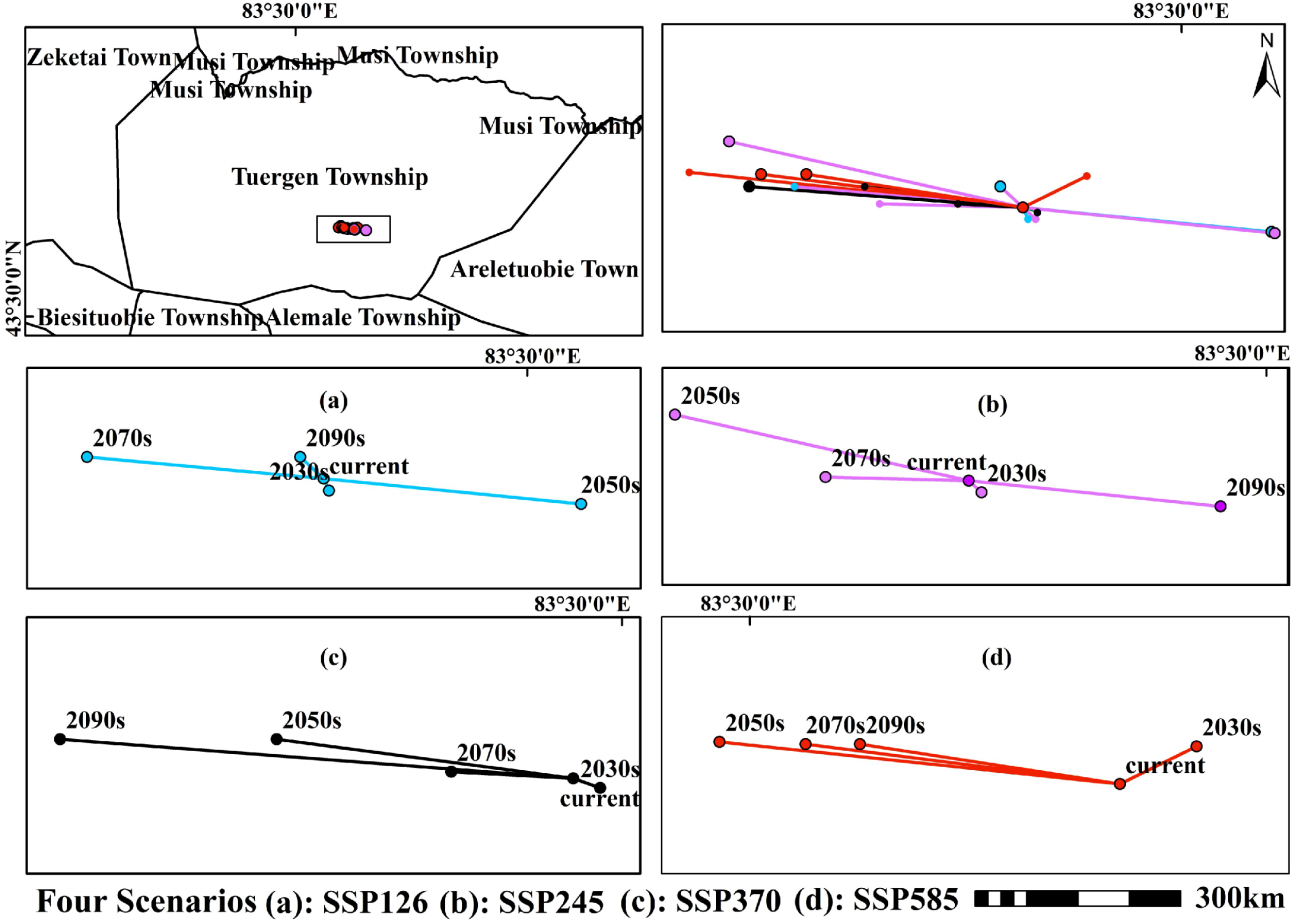
Centroid distribution of ragweed invasion under different climate scenarios.

In the context of future climate scenarios, **Figure 11** depicts the movement pattern of the potential distribution centroid of ragweed. Regardless of the climate scenario and projected period, the distribution centroid of ragweed consistently remained within Tuergen Township, Xinyuan County. Under different climate scenarios, the centroid exhibited distinct directional and displacement characteristics across all projected periods. Under the SSP126 scenario, centroid displacement was minimal in the 2030s (0.041 km toward the southeast, with a value of 157.4°). The centroid subsequently shifted eastward in the 2050s, with the largest displacement recorded under this scenario (0.617 km, bearing 88.6°). In the 2070s, the centroid reversed direction and migrated northwestward (0.566 km, bearing 298.1°), before shifting east–northeastward by 0.090 km (bearing 83.5°) in the 2090s. Under the SSP245 scenario, the smallest displacement was recorded in the 2030s (0.050 km toward the southeast, 135.0°), followed by the greatest displacement in the 2050s (0.756 km toward the northwest, 312.0°). In the 2070s, the centroid migrated northward (0.352 km, bearing 3.0°), and it then shifted eastward in the 2090s (0.625 km, bearing 89.6°). Under the SSP370 scenario, the centroid experienced the smallest displacement in the 2030s (0.039 km toward the southeast, with a value of 144.0°). Westward migration dominated the subsequent periods, with the centroid shifting west–southwestward in the 2050s (0.395 km, bearing 265.5°) and reaching the greatest displacement under this scenario in the 2090s (0.677 km, bearing 267.5°). A brief eastward deflection was observed in the 2070s (0.161 km, bearing 91.5°). Under the high-emissions SSP585 scenario, centroid migration demonstrated the strongest spatial response across all the scenarios. The centroid initially shifted north–northeastward in the 2030s (0.189 km, bearing 21.5°), followed by the maximum displacement recorded across all scenarios and periods in the 2050s (0.829 km toward the west–southwest, bearing 266.5°). Westward migration continued in the 2070s (0.642 km, bearing 270.0°) and 2090s (0.544 km toward the west–southwest, bearing 270.5°). Overall, the distribution centroid shifted toward the western and northwestern regions under most scenarios and periods, with the most pronounced spatial displacement occurring under the SSP585 scenario in the 2050s (0.829 km, bearing a value of 266.5°). This shift primarily resulted from reduced habitat suitability in low-elevation river valleys (as evidenced by the contraction of medium- to high-risk zones in **Figure 11**), passively displacing the distribution centroid rather than indicating large-scale colonization of extremely high-elevation regions by this species.

## 4 Discussion

### 4.1 Optimizing model performance

The results of this study, which are based on an improved MaxEnt model, indicate that the model for ragweed (*A. artemisiifolia* L.) is extremely accurate (AUC = 0.991) in Xinyuan County, demonstrating the high reliability of the model for predicting invasive plants at the county level. Previous studies have confirmed that appropriately adjusting the parameters of a MaxEnt model (FC combination and RM) can significantly increase the robustness and biological interpretability of the prediction results (Zhao et al., 2022). Compared with models with default parameters, parameter optimization can effectively mitigate overfitting and enhance a model’s responsiveness to complex climate–environment interactions (Cobos et al., 2019; Vignali et al., 2020). For example, in a study of *Quasipaa boulengeri*, an optimized MaxEnt model yielded results consistent with those of a random forest model, with AUC values exceeding 0.9 in both cases. These findings indicate that a parameter-tuned MaxEnt model can rival ensemble algorithms and demonstrate increased stability in areas with significant spatial heterogeneity (W. X. Zhao et al., 2022).

### 4.2 Driving mechanisms of the spatial distribution

The dominance of seasonal climate variables, rather than mean annual conditions, in shaping the ragweed distribution in Xinyuan County warrants interpretation. BIO15, BIO3, BIO4, and BIO12 together accounted for 68.3% of the cumulative contribution, indicating the importance of within-year climate variability over long-term averages in determining where this species can establish and persist. This conclusion is consistent with research on A. artemisiifolia’s worldwide niche, which revealed that BIO15 and BIO4 are the main factors affecting the spatial patterns of this species (Ki et al., 2022). Moreover, the significant contributions of temperature-related factors (BIO3 and BIO4) in this study also corroborate the findings that temperature variables dominate the large-scale distribution patterns of invasive plants in China (Ren et al., 2025). Notably (Ki et al., 2022), further predicted that the northward growth of this species will be influenced by future climate change, implying that control pressures in Xinyuan County and surrounding areas may intensify further with climate change.

Ragweed in this study area is primarily concentrated along road edges, reflecting the dominant roles of human disturbances and transportation networks in its spatial distribution (Müller et al., 2018). reported that linear infrastructure, such as highways and railways, serves as key ragweed habitat. Research in the Ili River Valley of the northwestern semiarid region (W. X. Zhao et al., 2022) revealed that ragweed accounted for as much as 27.96% of roadside vegetation cover, which was significantly greater than that in residential areas (10.75%) and grasslands (8.06%). This distribution pattern indicates that although ragweed currently occupies a low proportion of grassland habitats, the highway network acts as an “invasion corridor,” facilitating its penetration into natural grassland ecosystems. Frequent human disturbances and transportation infrastructure not only promote the colonization of ragweed along road embankments but also increase the risk of its spread into surrounding natural grasslands.

High-risk areas for ragweed invasion are concentrated primarily in mountain steppe and mid-low mountain meadow ecosystems. These grassland types are located predominantly at low to moderate elevations and are characterized by temperate climates with frequent human disruptions such as road construction and grazing. They exhibit low vegetation cover and loose community structures, readily creating vacant ecological niches that allow invading species to establish themselves and proliferate. These findings are consistent with those of (Khan et al., 2023), who reported significantly higher alien plant occurrence rates in low-elevation, highly disturbed areas than in high-elevation zones within semiarid mountain ecosystems. Environmental filtering and biotic resistance in alpine environments (W. Zhao et al., 2022) jointly constrain invasions. Similarly (Guido et al., 2024), reported that the primary drivers of grassland invasion are topography and human activity intensity, with open grasslands being most susceptible to invasion, whereas closed meadows demonstrate relatively high resistance. Additionally (Catford et al., 2019), reported that community susceptibility to invasion is closely linked to resource availability and disturbance frequency. High productivity and resource fluctuations promote the establishment of invasive species, whereas resource-limited communities exhibit low invasion risks. Mountain grasslands and low-to-mid-elevation montane meadows emerged as the primary susceptible habitats for ragweed invasion, showing a range of community vulnerability to the combined effects of geography, climate, and human activity. Notably, the low BIO12 optimum identified in this study likely reflects a valley-floor sampling effect rather than a county-wide physiological threshold, given that occurrence records were collected predominantly from low-elevation alluvial plains where gridded BIO12 values are substantially below the county mean.

### 4.3 Habitat evolution under future climate scenarios

At the county scale in Xinyuan County, in this study, the spatial patterns of possible acceptable habitats for ragweed (*A. artemisiifolia* L.) under present and future climatic conditions were determined based on an optimized MaxEnt model. The simulation results revealed that in the context of future climate scenarios, areas with high suitability ratings for ragweed will decrease significantly overall, whereas core suitable areas will maintain strong stability. This pattern aligns with the trend of habitat contraction revealed in numerous studies that relied on SDMs for climate change projections. Previous studies indicated that when predicting future species distributions, SDMs frequently project reduced spatial ranges of suitable habitats under climate warming scenarios. Such contraction is closely related to modeling methods, variable selection, and emission scenarios (Beaumont et al., 2016). For instance, researchers that predicted future distributions across multiple species have indicated that under numerous climate scenarios, the suitable ranges of species will either contract or undergo complex spatial rearrangements as environmental conditions shift. These findings likely reflect the constraining effect of climatic envelope conditions on species’ ecological niches (Beaumont et al., 2016).

It is projected that climate change will profoundly reshape the spatiotemporal patterns of invasive plant distributions. Existing large-scale simulation studies indicate that under warming scenarios, the climatically suitable ranges of invasive plants will undergo complex changes involving both contraction and expansion, leading to a spatial reorganization of invasion hotspots. For example, on the basis of distribution modeling predictions for 144 invasive plant species (Evans et al., 2024), reported that under a +2 °C warming scenario, the average invasion hotspots in the eastern United States will shift northeastward by approximately 213 km. Suitability is predicted to increase in some areas but decrease significantly in others, revealing the spatial heterogeneity of invasive plant distribution responses given the effects of climate change. Similarly, the MaxEnt-Kuenm model predicted that the suitable habitat for the invasive plant *Ulex europaeus* in the Colombian Andes would decrease by 12–31% (Ángel-Vallejo et al., 2024). However, influenced by its phenotypic plasticity and ability to expand its climatic niche, its distribution may extend to higher elevations. These findings take on additional meaning when considered alongside recent multispecies SDM work in which habitat suitability for four invasive alien plant species in Ethiopia was modeled under the CMIP6 SSP2-4.5 and SSP5-8.5 scenarios and reported substantial habitat expansion; *Prosopis juliflora*, for instance, was projected to increase its suitable range by up to 106.25% by 2070 under SSP5-8.5 (Yasin et al., 2026). This contrasts with the overall contraction projected for *A. artemisiifolia* in the present study, yet we suggest that the deviation reflects differences in regional climate trajectories and species-specific tolerance thresholds rather than a methodological inconsistency: Ethiopia’s warming lowlands may lead to new suitable space for drought-tolerant invasive areas, whereas the same warming trend in Xinyuan County may push local conditions beyond the tolerance ceiling of *A. artemisiifolia*. Notably, both studies converge on one key point, the spatial concentration of invasion risk, with high-risk zones clustered around specific landscape features in both cases, reinforcing the value of fine-scale spatial analysis for informing targeted management.

Consistent with these studies, the optimal MaxEnt model results for ragweed (*A. artemisiifolia*) in this research indicate a composite spatial dynamic pattern of “stable core—marginal contraction—local expansion” under future climate scenarios. Although the entire region that is suitable is projected to decrease, the core region is expected to remain stable and exhibit limited expansion toward higher elevations along river valleys, suggesting that the species may maintain its population by exploiting local microclimate refuges. These findings suggest that the impacts of future climate change on invasive plant risk will extend beyond overall habitat contraction, potentially manifesting as directional migration and vertical spatial redistribution. This prediction underscores the necessity of developing differentiated, spatially adaptive management strategies.

Research on the invasive plant *Ambrosia trifida* confirms that the “preference effect” of native species is key to suppressing invasions, sowing native plants six weeks before the invasive species can establish can significantly reduce the relative competitive index (RCI) of the invasive plant through resource preemption (Byun, 2023). Therefore, in terms of governance practices in Xinyuan County, it is recommended that highly competitive native forage grasses or indigenous plants be promptly reseeded after ragweed is physically removed to prevent secondary outbreaks of ragweed populations through ecological niche occupation. For the high-elevation expansion frontiers in the northwest predicted by the model, particularly areas bordering the Nalati Grassland Scenic Area, an early warning system should be established.

Interestingly, even though the distribution centroid exhibited predominantly westward and northwestward passive displacement under most future scenarios, the invasion risk for high-elevation grassland types did not significantly increase. This reveals an “ecological niche squeeze” effect facing ragweed under future climate conditions. On the one hand, warming climates cause the water–heat combination in low-elevation river valley core areas (mountain steppes) to exceed the optimal range for ragweed (managed as high-risk zones degrading to medium-risk zones); on the other hand, despite rising temperatures, the low-temperature barrier effect persists in high-elevation regions (mountain meadow grasslands), limiting its breakthrough into alpine zones. Thus, the passive displacement of the distribution centroid reflects the contraction of suitable habitat in low-elevation river valleys rather than active colonization of high-elevation zones by this species.

### 4.4 Limitations and future perspectives

Despite the insights gained from this study, several limitations should be acknowledged. First, the occurrence data acquired through field surveys are inherently constrained by terrain accessibility in mountainous regions. This logistical constraint introduced a nonrandom sampling pattern, with the highest point densities along road corridors and river valleys. Although we implemented spatial thinning with a minimum distance threshold of 25 km (equivalent to five times the 5-km grid resolution) to mitigate spatial autocorrelation, residual sampling bias could not be entirely eliminated. Crucially, however, road-associated variables were excluded from the environmental predictor suite, and the MaxEnt model was driven primarily by broad-scale climatic predictors (with BIO15, BIO3, BIO4, and BIO12 jointly accounting for 68.3% of the model contribution). This design substantially minimizes the risk of accessibility bias, artificially inflating the perceived importance of disturbance-related factors. Future studies could further refine these predictions by incorporating target-group background sampling or spatial bias information as regional biodiversity datasets become more comprehensive.

Second, future climate projections in this study rely on a single general circulation model (BCC-CSM2-MR). Although BCC-CSM2-MR was selected specifically for its optimized parameterization of East Asian monsoon dynamics and its widespread validation across China, aligning closely with the dominant climate drivers identified here, projections from a single GCM inevitably entail structural model uncertainty. We partially addressed this issue by evaluating four core emission scenarios (SSP1-2.6, SSP2-4.5, SSP3-7.0, and SSP5-8.5) to capture the full spectrum of radiative forcing trajectories. Nevertheless, constructing a multi-GCM ensemble remains highly desirable for better quantifying structural climate uncertainty and providing a more robust probabilistic assessment of *A. artemisiifolia* invasion risk in the future.

## 5 Conclusions

In this study, a Kuenm package-parameter-optimized MaxEnt model integrated with CMIP6 multiscenario climate models was used to systematically simulate the spatial and temporal progression of the potential habitat of *A. artemisiifolia* L. (common ragweed), a typical invasive plant in arid northwestern China, especially Xinyuan County. The key findings are as follows.

First, the parameter-optimized MaxEnt model accurately identified the ecological niche characteristics of invasive plants in areas of complex topography. The Kuenm package-optimized model effectively mitigated overfitting risk, achieving a training AUC of 0.991, a partial ROC ratio of 1.959 (*p* < 0.001), and an empirical omission rate of 0.0267, indicating its high reliability in predicting invasion risk at the county scale. The results of this study confirm that seasonal changes in water and heat conditions are the dominant ecological processes constraining ragweed colonization in Xinyuan County. Among these, BIO15 (23.1% contribution) and BIO3 (21.4% contribution) played decisive roles, revealing the high sensitivity of the species to uneven precipitation and variations in the amplitude of the diurnal temperature.

Second, the current spatial distribution of ragweed is characterized by pronounced “river valley–road network” clustering patterns and clear invasion preferences for specific grassland types. The simulation results indicate that high-risk zones are concentrated primarily in low-elevation river valley alluvial plains (e.g., Tuergen Township and Zeketai town) and are distributed in belts along major transportation corridors. Grassland type risk assessments indicate that mountain steppe and low–middle mountain meadow habitats, which are characterized by low elevations and high human disturbance intensities, constitute the primary vulnerable habitats for ragweed invasion. In contrast, high-elevation grassland types such as the mountain meadow steppe currently maintain extremely low invasion risk because of low-temperature environmental constraints.

Third, future climate change will drive structural alterations in ragweed distribution patterns, which will be characterized by “weakened suitability intensity and passive spatial displacement of the distribution centroid.” This study reveals that under multiple future scenarios from SSP126 to SSP585, the total area of ecosystems that are highly suitable for ragweed will show an overall decreasing trend. Specifically, the risk level in the original core area (mountain steppe) is predicted to significantly decrease from high to medium risk owing to excessive warming or moisture stress. The distribution centroid is projected to shift predominantly westward and northwestward under most scenarios, with the maximum displacement reaching 0.829 km under the SSP585 scenario in the 2050s; this shift primarily reflects passive displacement driven by the contraction of suitable habitat in low-elevation river valleys rather than active migration toward high-elevation areas. In addition, a limited expansion zone (7–16 km^2^) was identified penetrating toward higher elevations and upstream river tributaries under high-emission scenarios, representing an emerging invasion frontier that warrants vigilant monitoring. The low-temperature barrier effect of alpine ecosystems remains robust throughout the 21st century, limiting the breakthrough expansion of the species toward mountaintop regions.

Fourth, a “zonal prevention and control” strategy based on spatiotemporal heterogeneity is crucial for safeguarding regional ecological security. Given the potential threats of ragweed to agricultural production and human health, differentiated management is recommended. In river valley core zones and mountain grasslands, physical eradication should be combined with the replanting of native species to curb population outbreaks through interspecific competition mechanisms. In ecologically sensitive areas, particularly the Xinjiang Yili Wild Fruit Forest Nature Reserve, which is located around Alemale town, a “preventive containment” system should be established. To address the potential upward penetration of ragweed, priority monitoring should focus on the transitional zones between low-to-mid mountain meadows and alpine meadows to strictly prevent ragweed from establishing at higher elevations through grazing and tourism activities, thereby safeguarding regional ecological security.

## Data Availability

The raw data supporting the conclusions of this article will be made available by the authors, without undue reservation.
